# Neural networks for rapid phase quantification of cultural heritage X-ray powder diffraction data

**DOI:** 10.1107/S1600576724003704

**Published:** 2024-05-31

**Authors:** Victor Poline, Ravi Raj Purohit Purushottam Raj Purohit, Pierre Bordet, Nils Blanc, Pauline Martinetto

**Affiliations:** a Univ. Grenoble Alpes, CNRS, Grenoble INP, Institut Néel, 38000 Grenoble, France; b Univ. Grenoble Alpes, CEA, IRIG, MEM, NRS, 17 Rue des Martyrs, 38000 Grenoble, France; DESY, Hamburg, Germany

**Keywords:** neural networks, deep learning, X-ray diffraction, computed tomography, cultural heritage, tomography

## Abstract

Recent developments in synchrotron radiation facilities have increased the amount of data generated during acquisitions considerably, requiring fast and efficient data processing techniques. Here, the application of dense neural networks (DNNs) to data treatment of X-ray diffraction computed tomography (XRD-CT) experiments is presented. Processing involves mapping the phases in a tomographic slice by predicting the phase fraction in each individual pixel. DNNs were trained on sets of calculated XRD patterns generated using a Python algorithm developed in-house. An initial Rietveld refinement of the tomographic slice sum pattern provides additional information (peak widths and integrated intensities for each phase) to improve the generation of simulated patterns and make them closer to real data. A grid search was used to optimize the network architecture and demonstrated that a single fully connected dense layer was sufficient to accurately determine phase proportions. This DNN was used on the XRD-CT acquisition of a mock-up and a historical sample of highly heterogeneous multi-layered decoration of a late medieval statue, called ‘applied brocade’. The phase maps predicted by the DNN were in good agreement with other methods, such as non-negative matrix factorization and serial Rietveld refinements performed with *TOPAS*, and outperformed them in terms of speed and efficiency. The method was evaluated by regenerating experimental patterns from predictions and using the *R*-weighted profile as the agreement factor. This assessment allowed us to confirm the accuracy of the results.

## Abstract 2

A small, fast and efficient neural network for phase fraction prediction of X-ray diffraction big data is presented. A data-driven approach allows users to create their own training dataset, making the method fully customizable for each experience.

## 1. Introduction

Analysis of cultural heritage artifacts forces materials scientists to constantly innovate. Owing to the valuable nature of the objects, samples are usually difficult to obtain. Their fragility and uniqueness require non-destructive analysis to allow for further studies. In addition to these constraints, samples from pictorial works (paintings, frescoes, statues) are generally made up of multiple thin layers of pigments, resulting in a highly heterogeneous composition. To understand this stratigraphy in detail, high-resolution tomographic techniques are often necessary, which produce large amounts of data and require dedicated processing methods.

In light of these challenges, X-ray diffraction (XRD) is widely used as it is a non-destructive non-invasive technique that allows the user to identify and quantify crystalline compounds such as inorganic pigments. The past decade has seen the rise of high-resolution XRD computed tomography (XRD-CT) owing to the improved brightness of synchrotron radiation sources. This technique now allows us to identify and localize the phases inside ‘virtual’ tomographic slices of the sample at micrometre-size resolution without disrupting its integrity (Bleuet *et al.*, 2008 ▸). In XRD-CT, the sample is illuminated with a small X-ray beam, the beam size defining the resolution of the final tomographic reconstruction. The data are recorded by translating the sample perpendicular to the beam, and at each translation step, the sample is rotated along an axis perpendicular to the beam. In general, the 2D diffraction images are first integrated azimuthally to obtain 1D XRD patterns, which are easier to handle for further data processing such as phase identification or semi-quantification. In the end, the data are stored as a 3D matrix or data-cube of dimensions (*N*_t_, *N*_r_, *z*), where *N*_t_ is the number of translation steps, *N*_r_ the number of rotation steps and *z* the recorded 1D diffraction data. In order to obtain a sufficient spatial resolution with convenient powder averaging, the beam size (or the voxel dimension) is kept on the order of 10–20 µm. The data-cube is then transformed using tomographic reconstruction algorithms to provide, for each voxel in the sample virtual slice, an XRD pattern that can be analyzed to determine the phase content. For a typical micro-sample of around a hundred micrometres in size, a single tomographic slice acquisition can easily reach thousands of 1D patterns (*N*_r_ × *N*_t_). Processing this huge amount of data is a challenge in terms of time and complexity with conventional methods such as serial Rietveld refinement (Coelho, 2018 ▸) and is hardly feasible during the experiment.

One approach to dealing with such large amounts of XRD data was developed in the *XRDUA* software (De Nolf *et al.*, 2014 ▸). It is based on the decomposition of individual patterns into a sum of phases and batch fitting of the regions of interest associated with the identified phases, providing map phase distributions but not quantitative ones. Moreover, the processing can be time consuming and therefore not suitable for live data treatment. Over the years, machine-learning (ML) algorithms such as *k*-nearest neighbor (Altman, 1992 ▸), random forest (Tin Kam Ho, 1998 ▸) or support vector machine (Cortes & Vapnik, 1995 ▸) have also been applied to XRD data, achieving various analyses from crystal structure prediction (Oliynyk *et al.*, 2016 ▸) to phase identification (Bunn *et al.*, 2016 ▸). More recently, Bordet *et al.* (2021 ▸) managed to identify and quantify the crystalline phases from XRD-CT tomographic slices using multivariate analysis. The authors showed that non-negative matrix factorization (NMF) (Lee & Seung, 1999 ▸) is an efficient and fast tool to decompose the data-cube into a small set of physically meaningful components from which the voxel phase content can be determined.

Recent years have seen the rise of the application of another branch of ML algorithms to XRD data: deep learning (Omori *et al.*, 2023 ▸) and namely its convolutional neural networks (CNNs) (LeCun *et al.*, 2015 ▸). This emerging technique has generated significant interest in the materials science community for the processing of spectroscopic (Pouyet *et al.*, 2021 ▸) and/or scattering data (Choudhary *et al.*, 2022 ▸). For XRD applications, most studies have been limited to using CNNs and most of them as a classification problem (Wang *et al.*, 2016 ▸, 2017 ▸; Yang *et al.*, 2017 ▸; Park *et al.*, 2017 ▸; Ke *et al.*, 2018 ▸; Chitturi *et al.*, 2021 ▸; Purushottam Raj Purohit *et al.*, 2022 ▸; Assalauova *et al.*, 2022 ▸). For example, Lee *et al.* (2020 ▸) tried a multiple CNN architecture to perform phase identification on a quaternary compositional system and linked the predictions with three-step-phase-fraction quantification. Wang *et al.* (2020 ▸) used a data-driven approach, augmenting theoretical data to train a CNN for phase identification and demonstrated its supremacy over common ML algorithms. Lee *et al.* (2021 ▸) confirmed these results in another paper where their CNN for phase identification was followed by a dense neural network (DNN) for phase fraction prediction in regression mode. However, the overall procedure implies training more than a thousand DNNs for each case of a binary or ternary composition, making it system specific and time consuming. For regression problems, Dong *et al.* (2021 ▸) showed outstanding performance in predictions of scale factor, lattice parameter and crystallite size on a complex five-phase mixture system. Boulle & Debelle (2023 ▸) obtained promising results in the extraction of spatial strain profiles from XRD data of disordered irradiated materials.

In this work, we aim to establish a new method based on a DNN for phase fraction determination in XRD-CT data that is simple and fast enough for rapid and accurate data processing leading to phase quantification in the reconstructed voxels of the sample slice. For this purpose, we developed a data-driven protocol starting from phase identification and the Rietveld refinement of the sum pattern of the data-cube. This provides the initial parameters (identification of the phases and their microstructural properties), allowing a customized training of the DNN. Knowing that one of the main challenges of DNNs is to find enough training data similar to the real data, we built an in-house parallel processing Python algorithm able to generate hundreds of thousands of patterns within minutes. This data generator offers real flexibility to adapt the pattern-generation parameters to optimize the training dataset. A grid search optimization shows that the most efficient architecture of the DNN consists of a single hidden layer and thus its training time is reduced to minutes. We assess the method by regenerating patterns from predictions and comparing them with the experimental ones using agreement factors such as the *R*-weighted profile (*R*_wp_). The results from the DNN were also compared with serial Rietveld refinement using the *TOPAS* (Coelho, 2018 ▸) software. Here, we apply the analysis protocol to a mock-up and a historical sample of ‘applied brocade’, a sophisticated relief decoration designed to mimic the rich gold-embroidered fabrics worn by the nobility during the late medieval period (Geelen & Steyaert, 2011 ▸). The realization of such historical artifacts involved the superposition of several layers of different materials (metal sheets, beeswax, mineral pigments), which poses a real challenge in terms of analysis, as the samples undergo considerable degradation over time. The historical fragment has already been discussed extensively in a previous article by Bordet *et al.* (2021 ▸), allowing us to make a complementary evaluation of our method.

## 2. Experimental

### 2.1. Sample and XRD-CT measurements

**Figure 1:**
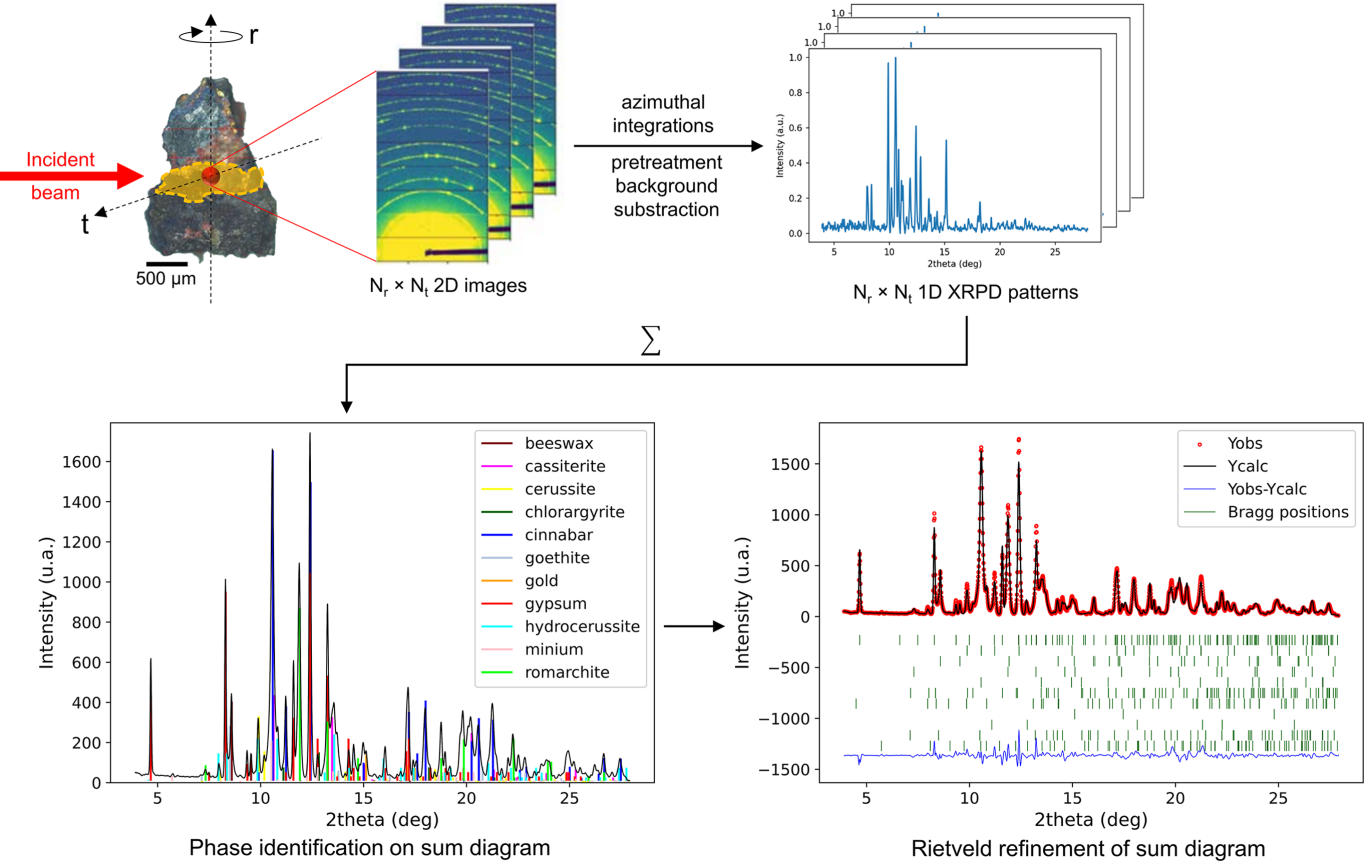
Workflow: from the experiment to the preliminary analyses for starting dataset generation.

The historical sample was taken from a wooden Pietà from the castle of Montrottier in Lovagny, France. Visual observations by a conservator were essential in identifying the presence of applied brocades and to clearly understand the succession of layers partially altered due to extensive overpaints. A preliminary *in situ* non-invasive study has been done using a portable X-ray fluorescence/XRD instrument which allowed a first stratigraphy of the decoration to be proposed (Martinetto *et al.*, 2021 ▸). The observed layer stacking is typical of an applied brocade where one finds, successively, a preparation layer, a priming paint layer, a filler material, a degraded tin foil, gilding and overpainting on top (Geelen & Steyaert, 2011 ▸). On the basis of these considerations, we fabricated a set of mock-ups on which the sequence of layers is well known in order to better evaluate the performance of our method. The historical sample used here is shown in Fig. 1 ▸ and has a roughly triangular platelet shape of around 1 mm × 200 µm × 50 µm (photographs of the mock-up can be found in Fig. S1 of the supporting information). The virtual slice analyzed inside the sample is shown in orange in Fig. 1 ▸.

The XRD-CT data were collected at the French CRG beamline BM02 of the ESRF (Grenoble, France). The experimental setup and the data preprocessing are detailed by Bordet *et al.* (2021 ▸) and partially summarized in Fig. 1 ▸. The 2D diffraction images were recorded with a 25 × 25 µm beam size, and then the contribution from a few single-crystal grains was filtered out and the resulting 2D patterns were azimuthally integrated using the *PyFAI* software (Ashiotis *et al.*, 2015 ▸). The shapes of our historical samples are often in the form of irregular platelets instead of an ideal cylinder and with a stratigraphy whose composition varies significantly from one layer to the next. Heavy elements may also be present in certain layers and, as we work at moderately high energies, their absorption can be significant. As a result, the background level and shape can fluctuate greatly during the tomographic experiment and cannot be properly taken into account by the DNN. A 1D rolling ball algorithm for background removal is applied to all individual patterns using carefully optimized parameters. Only the 2θ range showing valuable information is kept in order to reduce the volume of data (around 2000 observations per pattern). In the end, the data-cube contained 90 (*N*_r_) × 82 (*N*_t_) = 7380 1D patterns for the historical sample and 360 (*N*_r_) × 25 (N_*t*_) = 9000 for the mock-up.

The first step involves identifying the phases present in the sample virtual slice and characterizing their microstructure. Following the pre-treatment and background subtraction on the data-cube, we worked on the sum pattern (average of all data-cube patterns) using the *DIFFRAC.EVA* software (Version 5.2; Bruker AXS GmbH, Karlsruhe, Germany) and the PDF-2 2003 database (Gates-Rector & Blanton, 2019 ▸). For the mock-up, the three phases used in its manufacture were easily retrieved: anhydrite CaSO_4_ imitating the preparation layer; cinnabar, HgS, the priming paint layer; and romarchite, SnO, the degraded tin foil. In the historical sample, eleven phases were identified in the tomographic slice: beeswax, (CH_2_)_*x*_; cassiterite, SnO_2_; cerussite, PbCO_3_; chlorargyrite, AgCl; cinnabar; goethite, FeO(OH); gold, Au; gypsum, CaSO_4_·2H_2_O; hydrocerussite, Pb_3_(CO_3_)_2_(OH)_2_; minium, Pb_3_O_4_; romarchite. A Rietveld refinement was then carried out, keeping the atomic parameters fixed at their published values. An overall atomic displacement parameter was fixed for all the phases. The reflection profiles were described using the Thomson–Cox–Hastings (Thompson *et al.*, 1987 ▸) model from the refinement of an LaB_6_ standard and the sample-broadening effects for crystallite size. Only scale factors, unit cell and particle size parameters were refined. For each phase, a list of interplanar distances (*d*) and associated integrated intensities (*I*) was extracted from this refinement.

The data-cubes were then treated with serial Rietveld refinement performed using the *TOPAS* software (Coelho, 2018 ▸). The background was chosen to be constant and not refined. The same refinement file from the refinement of the sum pattern was used to refine all of the individual patterns the same way. For each phase, the refined parameters were the scale factors and the phase misplacement (particle size parameters were fixed at their values determined above).

### 2.2. Dataset generator and randomization strategies

It was clearly impossible to use experimental data as a training dataset for the DNN. It should have been built on the measurements of all identified phases mixed in different proportions with the same experimental resolution and setup. The phases should have a similar microstructure to that in the sample. Also, phases that are not located on the sample rotation axis yield a shift of the Bragg peak position in their powder pattern which varies with sample rotation. All these effects must be considered to accurately train the DNN to predict phase fractions. Therefore, we chose to generate a synthetic training dataset by calculating patterns, taking these effects into account.

**Figure 2:**
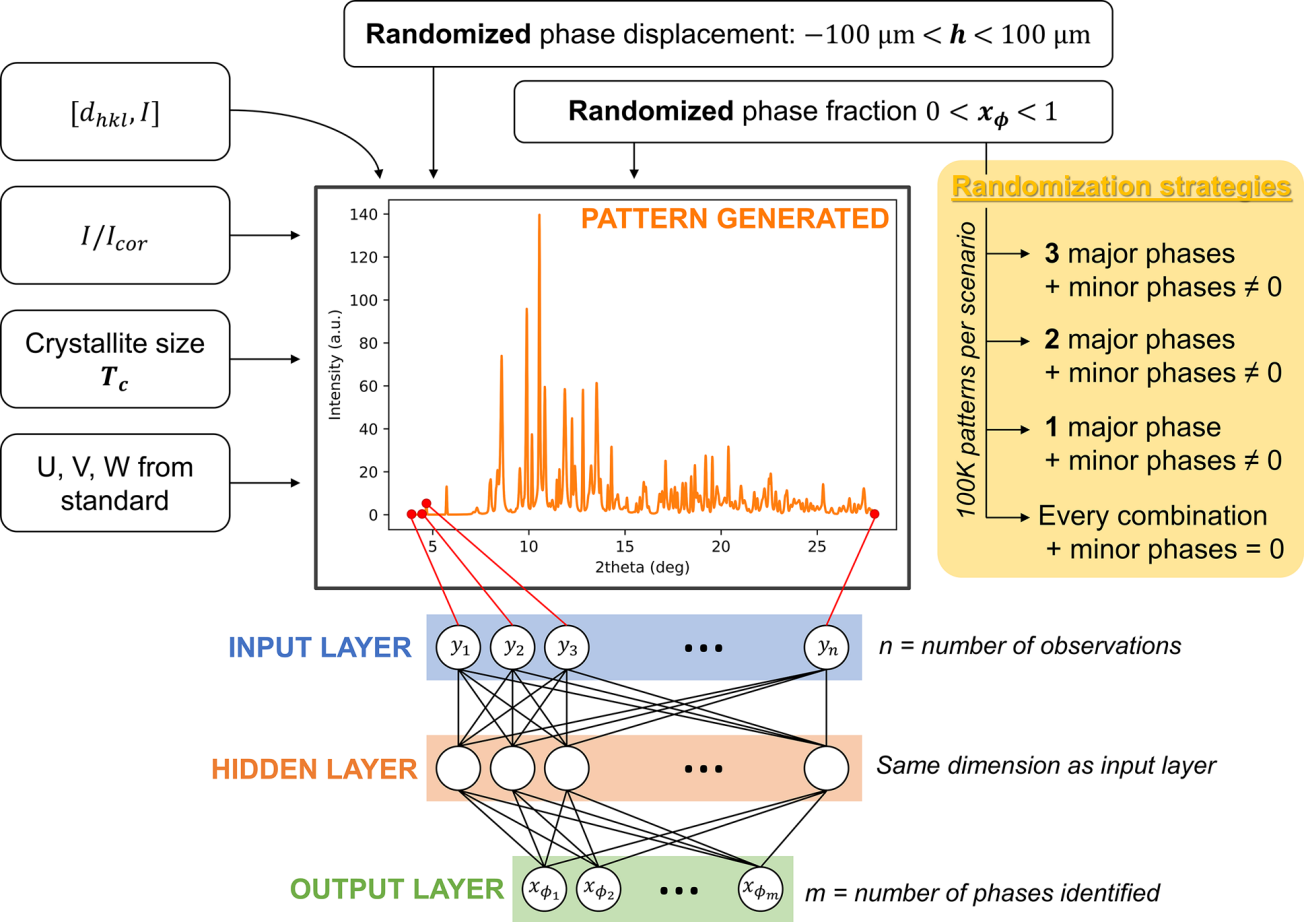
Dataset generation for training of the NN and architecture of the DNN.

A Python suite using parallel processing for fast execution was written to control the whole process from dataset creation to training and optimization of the DNN shown in Fig. 2 ▸. In the generation of the training dataset, each 1D XRD pattern of a given phase is characterized by a list of Bragg peaks that have several features, namely position, intensity, shape and width. For each phase, the position and intensity come from the Rietveld refinement of the sum pattern.

The quantitative analysis carried out in this study is based on the relative intensity ratio method detailed by Hubbard *et al.* (1976 ▸). It consists of calculating the proportions of each phase (*i*) in a pattern from its *I*_*i*_/*I*_cor_ ratio. This value has been defined as the ratio of the peak height of the strongest line of a sample to the strongest line of corundum (hexagonal reflection 113) for a 1:1 mixture, by weight, of the two phases. Note that we initially extracted *I*_*i*_/*I*_cor_ ratios directly from the pattern diffraction files of the PDF-2 2003 database, but the reported *I* values may be affected by differences in peak shape due to microstructure effects. Indeed, the approximation of intensity as the height of the strongest line and not its integrated intensity causes several inconsistencies in the *I*_*i*_/*I*_cor_ values within the PDF database. To avoid this problem, we decided to simulate theoretical patterns of a 1:1 mixture with corundum for each phase, using the list of *I* and *d* values obtained from the Rietveld refinement. More importantly, both phases were simulated using the same peak shape parameters from the refinement of the sum pattern, and the ratio was calculated using the integrated intensities.

The lists of interplanar distances and associated intensities, together with the *I*_*i*_/*I*_cor_ ratios obtained from pre-processing the sum pattern, provide the initial peak positions and intensities. However, positions can be affected by sample displacement when the corresponding phase does not lie on the rotation axis. This problem, known as the parallax artifact, is common in XRD-CT experiments and has been described thoroughly in previous work (Scarlett *et al.*, 2011 ▸; Rowles & Buckley, 2017 ▸; Stock *et al.*, 2019 ▸; Vamvakeros *et al.*, 2020 ▸). It results in a shift in Δ2θ of the peaks along the 2θ axis which we describe as 

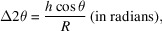

where *R* is the distance between the sample and the detector (Klug & Alexander, 1974 ▸); *h* is the positional shift of the phase, and will be a variable parameter of the dataset generation, randomized between −100 to +100 µm for our samples of 200 µm maximum size.

The Bragg peak profiles were treated as pseudo-Voigt functions. Regarding the peak widths, we used the Thomson–Cox–Hastings model (Thompson *et al.*, 1987 ▸) with independent FWHMs for the Gaussian and Lorentzian components to separate instrumental (Gaussian) and sample effects (Lorentzian). We also assumed that the peak broadening from the sample was only due to domain size effects and not strain. This hypothesis was confirmed by the Rietveld refinement of the sum pattern. Hence, the FWHMs *H*_G_ and *H*_L_ of a peak can be written as a function of the Bragg angle using Cagliotti’s law and Scherrer isotropic broadening: 

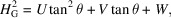




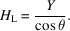

The values of *U*, *V* and *W* represent the instrument resolution function and are obtained by Rietveld refinement of an LaB_6_ standard sample pattern as mentioned above. Considering an isotropic model, the Scherrer equation allows us to relate the size of the crystallites (*T*_c_) to the broadening of a peak in a diffraction pattern as follows: 

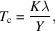

where *K* ≃ 0.9 and λ is the wavelength used for the experiment (here λ = 0.62 Å). For generation of the training dataset, the *T*_c_ value of each phase was fixed at the value determined by the Rietveld refinement of the sum data. Finally, the peak profiles were calculated as a pseudo-Voigt, PV = ηL + (1 − η)G, of FWHM *H* and mixing parameter η calculated from *H*_G_ and *H*_L_ using the Thomson–Cox–Hastings law (Thompson *et al.*, 1987 ▸).

On the basis of the above assumptions, the list of *d* and *I* values, the values of *T*_c_ and *h*, and the *U*, *V* and *W* parameters, one can construct the diffraction pattern for a single phase corresponding to the actual measurement setup. To build the training dataset, we now need to generate a large number of patterns representing the possible distribution of the phase fraction (*x*_ϕ_) over the measured data-cube. To obtain a varied training dataset close to the real data, we need to define a randomization strategy to cover as many cases as possible. Firstly, given our layered structure and beam size, we assume that, in a single pattern, there can only be a maximum of three phases in significant proportions, known as ‘major’ phases, the rest being zero or considered ‘minor’ phases. For the historical sample of eleven phases, we consider a first scenario in which we generate patterns with three ‘major’ phases among the eleven identified. They are generated in greater proportion than the eight others which are then considered minor phases and have much lower proportions [ 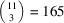 possible combinations]. We then repeat this scenario, this time considering only two major phases and the nine others as minor phases [ 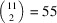 possible combinations] and then with only one major phase [ 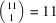 possible combinations]. In order to fill in the missing possibilities and provide a full range of phase fractions for NN training, we consider a final scenario in which all combinations of one to eleven phases are possible, *i.e.* 
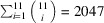
 combinations (here, the ‘minor’ phases are equal to 0). For each scenario, we created around 100 000 XRD patterns by varying the values of *h* and *x*_ϕ_, yielding a dataset of 399350 patterns of 2150 points each (same step size as the experimental ones). The whole dataset was generated in around 25 min on 40 cores of the ESRF network. The training 2D array dataset stores the phase fractions, crystallite size and sample displacement that generate each individual pattern resulting in a total shape of (399350, 2150 observations + 11 phases × 3 parameters = 2183) and 6.5 Gb size. For the mock-up, the same strategy can be adopted, but it can also be reduced, as it is easier to obtain a variety of cases for a three-phase system. A synthetic representation of the overall method is shown in Fig. 2 ▸.

### 2.3. Neural network architecture and training setup

First, in order to optimize the NN, we performed a grid search on a simple theoretical three-phase system similar to our mock-up sample (anhydrite, cinnabar and romarchite) with all hyperparameters variable: dropout, optimizer learning rate, activation and output functions, batch size, and number of epochs. For the network architecture, the number of layers was limited to 1, 2 or 3 hidden layers with a number of neurons that is a multiple of the number of observations in the input patterns (here, 2150 observations). The three systems tested were a network with one hidden layer of 2150 neurons; with two hidden layers of 2150 neurons each; and with three hidden layers with one layer at 2150, a second at 2 × 2150 = 4300 neurons and a final layer at 2150 neurons. Each network had an input layer of 2150 neurons and an output layer of three neurons corresponding to the three phase fractions to be predicted. The measure for grid search was the negative mean absolute error (NMAE), and the performance of each run was fivefold cross-validated on a dataset of 400 000 patterns normalized by their maximum intensity (80% training and 20% validation with no overlap between the two). This first optimization allowed us to fix most of the hyperparameters. Thus, a hyperbolic tangent was used as an activation function for the hidden layers and a rectified linear unit (ReLU) was used as the output function, with no dropout between layers, as this did not improve overall performance. Note that the ReLU activation function for hidden layers performed almost as well as the hyperbolic tangent. The loss function was the mean absolute error (MAE) with the mean squared error (MSE) as metric. Adam was used as an optimizer with a learning rate of 0.001. The DNN was trained over 20 epochs with a batch size of 256 patterns. Callbacks were implemented to save the best model and to stop training when no significant learning was observed on the loss function after 5 epochs. Training results for different architectures can be found in Figs. S2–S4 and Table S1 of the supporting information.

This first grid search showed that single-layer networks seem to outperform the other two systems considered, and that increasing the number of parameters (weights and biases) to be learned, in addition to the considerable increase in learning time, does not improve performance. To confirm this result, a further grid search was carried out using the hyperparameters described above, with a focus on varying the number of parameters to be learned consistently. Considering the same three networks as before, we applied a ratio to the number of neurons per hidden layer to check the evolution of negative MAE versus the number of parameters (see Fig. S5). The results show that the optimum is reached on the single hidden layer system and when the number of neurons in the network is equal to the number of observations. A similar simple DNN architecture was used by Lee *et al.* (2021 ▸), who employed a CNN for phase identification. The authors noted that, as the DNN architecture became deeper, the model performance deteriorated considerably due to the lack of training data. In our case, even though the amount of training data was less limited, deepening the architecture increased training time without significantly improving performance, and even sometimes deteriorating it. Fig. 2 ▸ shows the architecture that consists of a first layer of input neurons, where each observation of the pattern is fully connected to the hidden layer, the size of the input layer being the same as that of the hidden layer. The size of the output layer depends on the number of phases to be identified, with each neuron giving the fraction of the corresponding phase. In the end, the total time of training of this kind of network does not exceed 5 min on a basic personal computer.

## 3. Results and discussion

### 3.1. Phase fraction determination and mapping

We decided to work on the experimental patterns before tomographic reconstruction, as the algorithms used for reconstruction can significantly affect the patterns and make their processing inaccurate (examples and results on the patterns from the reconstruction for the historical sample can be found in Figs. S8 and S9). Like the patterns used for training, the experimental patterns were normalized to their maximum intensity before being processed by the network. For the mock-up, the phase fractions of the whole data-cube of 9000 patterns are predicted in less than 1 s. The results of the total semi-quantification are presented in Table 1 ▸ together with the results of the sum pattern quantification and those obtained from the serial Rietveld refinement of all individual patterns. Note that the DNN results are consistent between trainings with a mean standard deviation of 0.3% for five distinct trainings. Overall, we find the same trends emerging here between the three methods, *i.e.* a large majority of anhydrite and the other two phases in smaller proportions. The first two methods appear to agree on the anhydrite and cinnabar fractions, whereas the romarchite fraction is identical between the DNN and the Rietveld refinement of the sum pattern.

**Figure 3:**
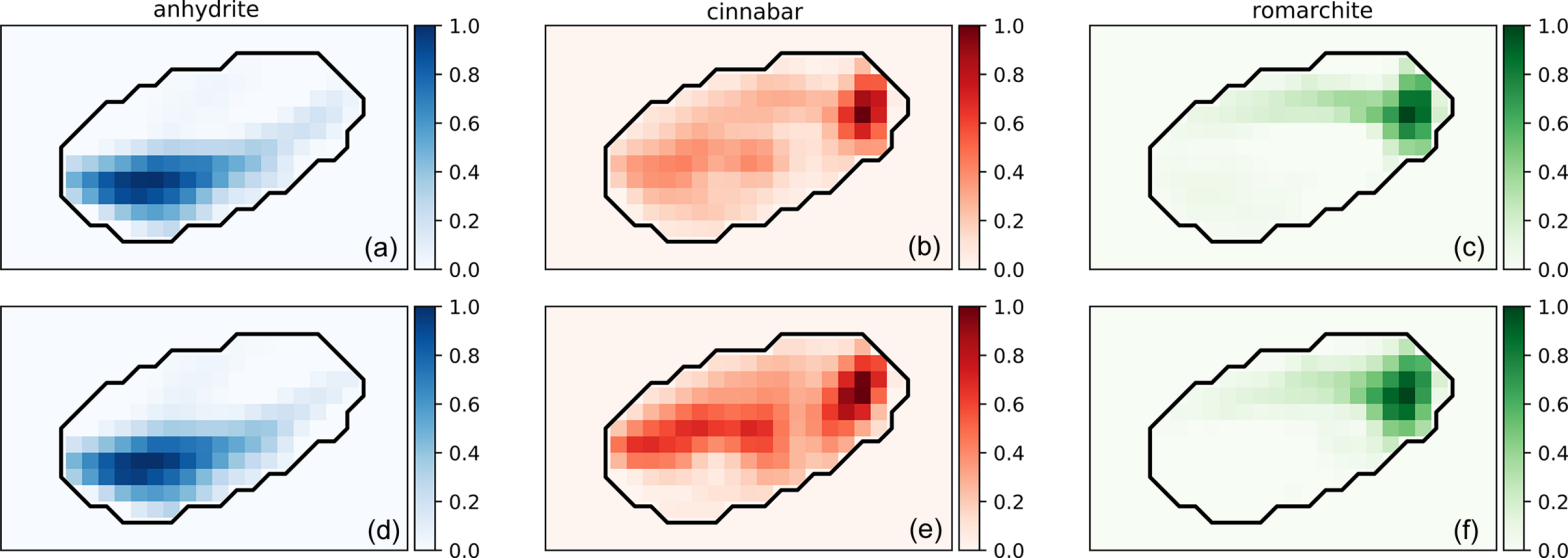
Phase fraction maps of the three phases of the mock-up predicted by (*a*)–(*c*) the DNN and (*d*)–(*f*) serial Rietveld refinement. The scale is given in arbitrary units for comparison between the two methods. The approximate shape of the sample is shown as a black line. Maps without normalization are available in Fig. S6.

As a result of the semi-quantification of the individual patterns, we obtain a data-cube of *N*_r_ × *N*_t_ × 3, the third dimension being the fractions of the three phases in each pattern for the serial Rietveld refinement and the DNN. This then allows us to map these phases to see their distribution in the tomographic slice. For this purpose, the data-cube is transformed into a reconstructed tomographic slice using the iradon (inverse radon transform) function of the *scikit-image* Python library (Van der Walt *et al.*, 2014 ▸). Fig. 3 ▸ shows the results for DNN and serial Rietveld refinement. The anhydrite and romarchite maps appear to be in good agreement, whereas the cinnabar map differs in certain areas in the middle of the tomographic slice. As mentioned above and shown in Table 1 ▸, the DNN appears to underestimate the amount of cinnabar in some of the patterns, resulting in a mismatch in the reconstructions.

Following these satisfying results, we applied the same method for the historical sample and the results are shown in Table 2 ▸. The same consistency in the DNN results is observed here with a mean standard deviation of 0.08% for five distinct trainings. Interestingly, the DNN and the serial refinement appear to match each other better than they match with the others (average absolute difference of 2.4% between fraction phases versus 4.7% for sum and 6.9% for NMF), confirming that an individual treatment of patterns differs from an analysis of the sum pattern or global multivariate analysis as used with NMF. One of the main differences lies in the quantification of the beeswax, a problem already identified in the previous study where accurate estimation seems difficult to achieve as the phase presents only two observable Bragg peaks in the diffraction pattern (Bordet *et al.*, 2021 ▸). The structure of the beeswax is not precisely known and is identified as ‘*n*-paraffin’ (PDF-49 1995) in the PDF database. The method to include beeswax intensities measured using modern samples in the reference intensity ratio quantification process is described by Bordet *et al.* (2021 ▸). However, experimental intensities can vary significantly from the reference due to the wax preparation (heating, spreading, cooling) or differences in the nature of the beeswax used by the medieval craftsmen. Moreover, the network had less ease in finding accurate predictions for gypsum. A possible source of error may be the distribution of phase fractions in the training dataset. In the historical sample, beeswax and gypsum are often present alone and not mixed with other phases, leading to experimental patterns of almost pure gypsum or beeswax (*i.e.* their phase fraction is around 1). The training dataset is probably devoid of this type of pattern, and the network is therefore not sufficiently trained for these cases. Finally, gypsum is present mainly at the sample boundary, where the total pattern signal can be affected. This makes background subtraction more prone to inaccuracy and can therefore lead to poor prediction of phase fractions.

**Figure 4:**
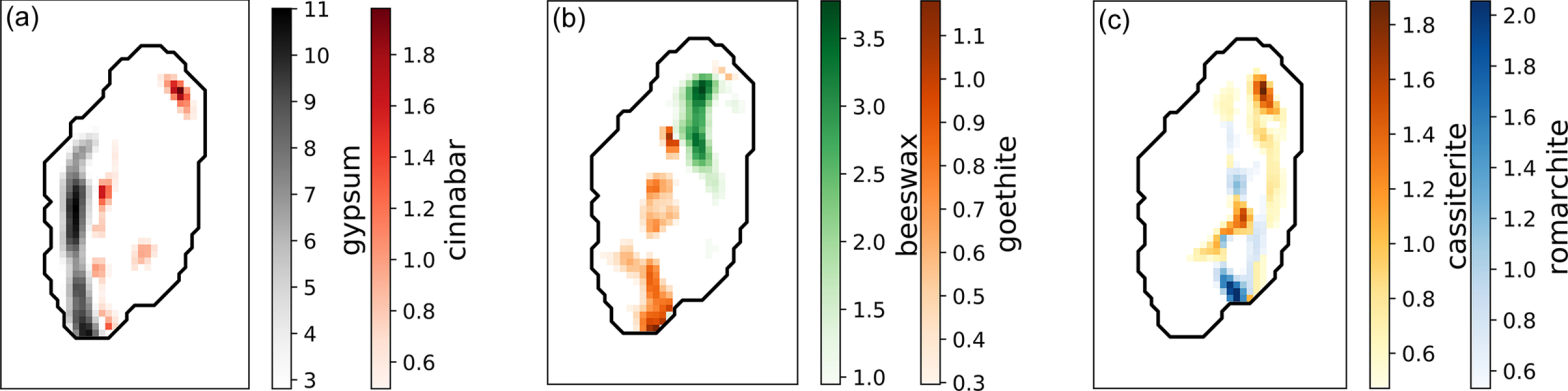
Summary of the phase fraction mapping of the main phases for each theoretical layer of an applied brocade: (*a*) preparation layer, (*b*) filler layer and (*c*) tin foil. For each slice, only two phases are considered and, on each pixel, only the most abundant phase is represented. For clarity, the bottom of the scale starts at 25% of the maximum value. The approximate shape of the sample is shown as a black line.

Like for the mock-up, we can map the eleven-phase distribution in order to go back to the layer sequence of the applied brocade. Fig. 4 ▸ presents three tomographic phase maps corresponding to the layers of applied brocades described above. From left to right:

Layer (*a*) is the preparation layer applied to the statue before the decorations are placed on it. It is made of gypsum and a priming paint layer made of cinnabar.
Layer (*b*) corresponds to the filler material used to ease the manipulation of the decorations and their application on the object. In this case, it is made of a two-layer structure of beeswax and goethite already observed during *in situ* analyses (Martinetto *et al.*, 2021 ▸).
Layer (*c*) corresponds to the surface of the applied brocade and the statue where the decoration was visible. This is usually gold applied with a layer of gilding on tin leaf. Here, the amount of gold is too small to be represented (a map is available in the supporting information). However, we were able to map the two tin oxides, romarchite and cassiterite, which reveal the shape and location of the degraded tin foil. A similar result for fully degraded tin was already observed on other statues of the same corpus (Poline *et al.*, 2023 ▸).

The results of the phase mapping are consistent with a classic applied brocade structure (Geelen & Steyaert, 2011 ▸). In particular, the DNN approach seems to achieve an accurate representation of the stratigraphy, mainly for the distribution of the two tin oxides and for the cinnabar of the priming paint layer. Note that the cinnabar spots away from the gypsum in Fig. 4 ▸(*a*) can be attributed to overpainting and are therefore not part of the original stratigraphy. The cerussite and hydrocerussite maps in Fig. S7 are superimposed on these spots, confirming a lead white and vermilion-based overpainting.

### 3.2. Method assessment

Compared with other methods used on the same sample, the DNN results seem to be generally rather accurate with regards to semi-quantification, both with global methods (sum pattern, NMF) and with those which process individual patterns (serial Rietveld refinement). The phase fraction maps also reveal that the results obtained with DNN are consistent with the sequence of layers expected for an applied brocade. However, absolute evaluation of the method remains difficult. A common evaluation method is to extract a few individual patterns and observe the match between experimental and calculated patterns.

**Figure 5:**
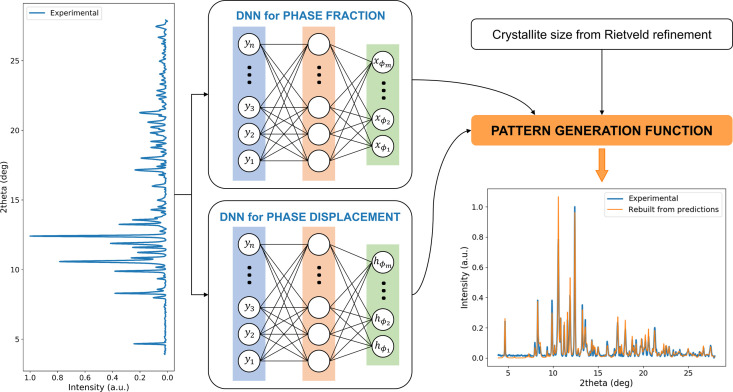
Schematic representation of the pattern regeneration. Phase fraction and sample displacement predictions are performed on two separate DNNs of similar architecture. The crystallite sizes are the same as those used in the dataset generation (extracted from the Rietveld refinement of the sum pattern). A scaling factor is applied for comparison between the reconstructed and experimental patterns.

**Figure 6:**
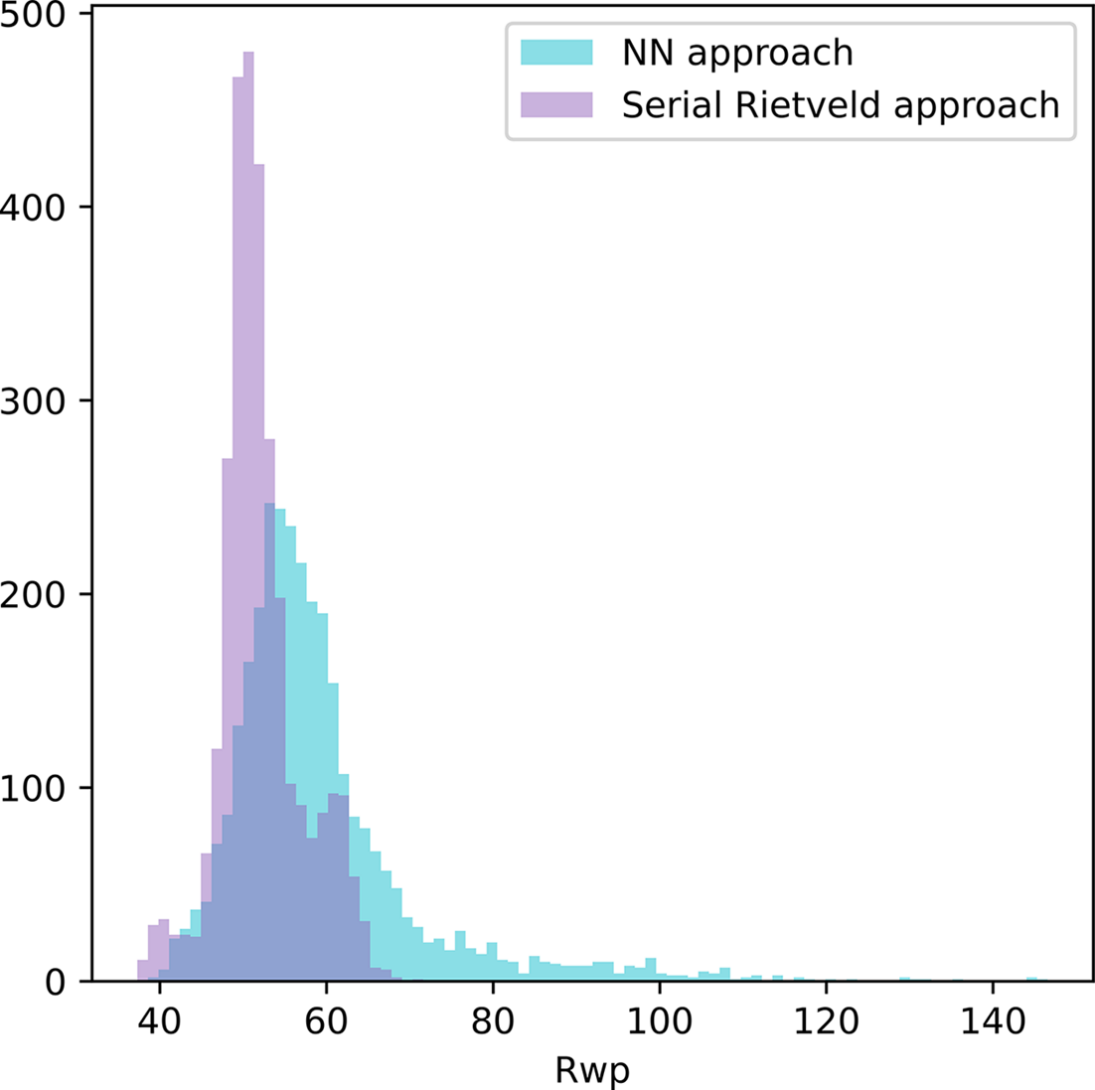
Histogram representations of *R*_wp_ for all the patterns (some outliers are not represented for a better readability of the classes, nine in total) from NN predictions and from serial Rietveld refinement.

Following this idea, we need to reconstruct the patterns using the results of the DNN process in order to calculate their agreement factor. In order to reconstruct the patterns, we can use the values of the *U*, *V* and *W* parameters for the instrumental resolution, the list of [*d*_*hkl*_, *I*] and *T*_c_ for each phase, and the phase fractions *x*_ϕ_ obtained by applying the DNN discussed above. However, the values of the sample displace­ments (*h*), although introduced as randomized parameters in the training dataset, are not extracted from the DNN and thus remain unknown. In order to obtain the values of *h*, we added another DNN of similar hyperparameters and architecture (except the learning rate of the optimizer is fixed at 0.01 instead of 0.001) to the process. This DNN operates independently from the phase fraction one, as depicted in Fig. 5 ▸. Note that Dong *et al.* (2021 ▸) used a similar method but on a complex architecture CNN linked with three DNNs in parallel: one for the prediction of the scale factor, one for the crystallite size and one for the lattice parameter. However, optimizing and training this type of network is quite time consuming, whereas here, with a training session lasting less than 5 min, we are not limited in the number of networks we can use. Moreover, having two distinct networks simplifies the DNNs’ learning and can improve their performance. The performance of this second DNN to extract phase displacements seems somewhat poorer compared with that for phase fractions (plots available in Fig. S10), probably due to the smaller effect of sample displacement on such complex patterns. Nevertheless, the predictions obtained are sufficient for us to rebuild the patterns. For the historical sample, 7380 reconstructed patterns are generated in around 3 min using the same parallel processing function described above. In order to compare the patterns (experimental and reconstructed), we use *R*_wp_ defined as follows: 

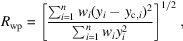

with *y*_*i*_ being the experimental observation, *y*_c,*i*_ the calculated observation and *w*_*i*_ the weight of each observation (here, in Poisson statistics: *w*_*i*_ = 1/*y*_*i*_). The *R*_wp_ histograms of the DNN compared with the serial Rietveld refinement are displayed in Fig. 6 ▸ and show good agreement between the two methods with slightly better results for the serial Rietveld refinement (*R*_wp_ histogram for the mock-up available in Fig. S11). Note that the high values of *R*_wp_ are due to the background subtraction which modifies the counting statistics and greatly decreases the denominator value in the *R*_wp_ calculation.

**Figure 7:**
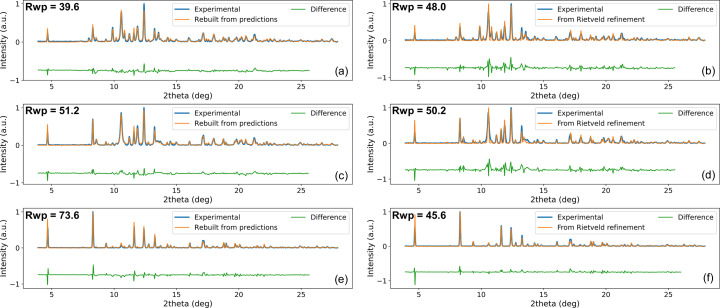
Left: three examples of experimental patterns (blue) compared with their rebuilt patterns (orange) for low, medium and high *R*_wp_ values. The difference between the two patterns is plotted in green. Right: the same examples with serial Rietveld refinement.

Fig. 7 ▸ shows a comparison between experimental and reconstructed patterns for selected *R*_wp_ values for both methods. For the low-*R*_wp_ examples [Figs. 7 ▸(*a*)–7 ▸(*d*)], the reconstructed pattern corresponds precisely to the experimental pattern, supporting the conformity of the results from the DNN method. Moreover, for the first two examples, even though the *R*_wp_ values are comparable for the two methods, it seems that the DNN predictions better match the experimental data. With regards to the high-*R*_wp_ example for the DNN [Fig. 7 ▸(*e*)], the integrated intensities of the rebuilt pattern appear to be broadly in line with experimental intensities. However, the mismatch is mainly due to the position of the peaks and therefore to the poor prediction of the phases’ displacement by the second DNN. In comparison, the example from the serial Rietveld refinement [Fig. 7 ▸(*f*)] seems to be in better agreement with the experimental pattern due to better phase displacement determination. This result has been observed for a number of other patterns, meaning that a high *R*_wp_ value is not necessarily linked to poor prediction of phase fractions and therefore supports the accurate results of the DNN method. Note that, despite some poor predictions of phase displacements, rebuilding the patterns from these predictions always leads to better results than for reconstructed patterns without sample displacements (Figs. S12 and S13 show *R*_wp_ histograms considering no sample displacement and sample displacement from serial Rietveld analysis).

## 4. Conclusions

This study demonstrates the suitability of DNNs for real-time processing of very large datasets from XRD-CT acquisitions. These DNNs have a basic architecture with a single hidden layer sufficient to accurately predict the phase fractions of complex systems such as highly degraded multi-layered cultural heritage samples. As the difficulty with this type of algorithm lies in the lack of training data, we created our own XRD pattern generation function using an XRD pattern description similar to the one used in the Rietveld method. In order to improve this description, we have chosen a data-driven approach to better match the generated patterns with the experimental ones. It is based on the Rietveld refinement of the sum pattern, which allows us to create our own lists of interplanar distances and integrated intensities, while giving us other key information such as the size of diffracting domains. After a grid search optimization on a basic theoretical three-phase system, we applied the DNN on a mock-up and a historical sample. The approach provides satisfactory results, similar to other methods used on the same sample. The phase fraction maps show the expected succession of layers that can be found in this type of cultural heritage sample, confirming the performance of our method. In addition, we also evaluate our method through agreement factors between experimental patterns and patterns regenerated from predictions, enabling us to confirm the accuracy of this data-driven approach. Although certain assumptions were made in carrying out this work, it seems that the quality of the results obtainable in such a short amount of time may be an essential help in the time-limited environment of a synchrotron experiment. Its ease of use and speed of execution are assets that allow scientists to easily adapt manipulations and thus save invaluable research time. Although it has been developed for XRD-CT data, the same method can be applied to other massive data collection techniques where the proportions of phases with constant structure vary over space (*e.g.* mapping) or time (battery cycling *etc.*).

## 5. Code environment and data availability

Everything was developed on JupyterLab (Version 3.4.0) and Notebook (Version 6.4.11) using ESRF computing resources (1 CPU of 40 cores). The packages were used in the following versions: tensorflow = 2.11.0, h5py = 3.6.0, matplotlib = 3.4.3, numpy = 1.21.6, scikit-learn = 1.0.2. The codes developed in this study can be found at https://github.com/polinev/NNXRD-mfraction and the data to run the codes at https://zenodo.org/records/10958419. 

## Supplementary Material

Supporting figures and table. DOI: 10.1107/S1600576724003704/yr5124sup1.pdf

## Figures and Tables

**Table 1:** Phase fractions (%) for the whole virtual sample slice of the mock-up of the three phases obtained by Rietveld refinement of the sum pattern, serial Rietveld refinement and the DNN For DNN predictions, the results are an average of five different trainings.

Phases	Rietveld sum	Serial Rietveld	DNN
Anhydrite	86.8	85.4	93.3
Cinnabar	10.3	9.0	3.9
Romarchite	2.9	5.6	2.8

**Table 2:** Phase fractions (%) for the whole virtual sample slice of the historical sample of the eleven phases obtained by Rietveld refinement of the sum pattern (Bordet *et al.*, 2021 ▸), NMF (Bordet *et al.*, 2021 ▸), serial Rietveld refinement and the DNN For DNN predictions, the results are an average of five different trainings.

Phases	Rietveld sum	NMF	Serial Rietveld	DNN
Beeswax	40.9	57.5	18	20.9
Cassiterite	14.6	10.9	8.6	9.7
Cerussite	0.9	0.5	1.8	0.7
Chlorargyrite	1.0	0.5	0.9	0.5
Cinnabar	7.8	1.3	11.9	9.4
Goethite	1.9	1.8	3	6.6
Gold	0.2	0.2	0.6	0.3
Gypsum	23.1	18.0	37	42.7
Hydrocerussite	1.3	1.6	4.6	1.3
Minium	<0.01	0.1	1.4	<0.01
Romarchite	8.3	7.6	12.2	7.9

## References

[bb1] Altman, N. S. (1992). *Am. Stat.* **46**, 175–185.

[bb2] Ashiotis, G., Deschildre, A., Nawaz, Z., Wright, J. P., Karkoulis, D., Picca, F. E. & Kieffer, J. (2015). *J. Appl. Cryst.* **48**, 510–519. 10.1107/S1600576715004306 PMC4379438 25844080

[bb3] Assalauova, D., Ignatenko, A., Isensee, F., Trofimova, D. & Vartanyants, I. A. (2022). *J. Appl. Cryst.* **55**, 444–454. 10.1107/S1600576722002667 PMC9172041 35719305

[bb4] Bleuet, P., Welcomme, E., Dooryhée, E., Susini, J., Hodeau, J.-L. & Walter, P. (2008). *Nat. Mater.* **7**, 468–472. 10.1038/nmat2168 18425135

[bb5] Bordet, P., Kergourlay, F., Pinto, A., Blanc, N. & Martinetto, P. (2021). *J. Anal. At. Spectrom.* **36**, 1724–1734.

[bb6] Boulle, A. & Debelle, A. (2023). *Mach. Learn. Sci. Technol.* **4**, 015002.

[bb8] Bunn, J. K., Hu, J. & Hattrick-Simpers, J. R. (2016). *JOM*, **68**, 2116–2125.

[bb9] Chitturi, S. R., Ratner, D., Walroth, R. C., Thampy, V., Reed, E. J., Dunne, M., Tassone, C. J. & Stone, K. H. (2021). *J. Appl. Cryst.* **54**, 1799–1810. 10.1107/S1600576721010840 PMC8662964 34963768

[bb10] Choudhary, K., DeCost, B., Chen, C., Jain, A., Tavazza, F., Cohn, R., Park, C. W., Choudhary, A., Agrawal, A., Billinge, S. J. L., Holm, E., Ong, S. P. & Wolverton, C. (2022). *npj Comput. Mater.* **8**, 59.

[bb11] Coelho, A. A. (2018). *J. Appl. Cryst.* **51**, 210–218.

[bb12] Cortes, C. & Vapnik, V. (1995). *Mach. Learn.* **20**, 273–297.

[bb13] De Nolf, W., Vanmeert, F. & Janssens, K. (2014). *J. Appl. Cryst.* **47**, 1107–1117.

[bb14] Dong, H., Butler, K. T., Matras, D., Price, S. W. T., Odarchenko, Y., Khatry, R., Thompson, A., Middelkoop, V., Jacques, S. D. M., Beale, A. M. & Vamvakeros, A. (2021). *npj Comput. Mater.* **7**, 74.

[bb15] Gates-Rector, S. & Blanton, T. (2019). *Powder Diffr.* **34**, 352–360.

[bb16] Geelen, I. & Steyaert, D. (2011). *Imitation and Illusion: Applied Brocade in the Art of the Low Countries in the Fifteenth and Sixteenth Centuries.* Brussels: KIK-IRPA Royal Institute for Cultural Heritage, Scientia Artis 6.

[bb17] Hubbard, C. R., Evans, E. H. & Smith, D. K. (1976). *J. Appl. Cryst.* **9**, 169–174.

[bb18] Ke, T.-W., Brewster, A. S., Yu, S. X., Ushizima, D., Yang, C. & Sauter, N. K. (2018). *J. Synchrotron Rad.* **25**, 655–670. 10.1107/S1600577518004873 PMC5929353 29714177

[bb19] Klug, H. & Alexander, L. (1974). *X-ray Diffraction Procedures: For Polycrystalline and Amorphous Materials.* New York: Wiley.

[bb20] LeCun, Y., Bengio, Y. & Hinton, G. (2015). *Nature*, **521**, 436–444. 10.1038/nature14539 26017442

[bb21] Lee, D. D. & Seung, H. S. (1999). *Nature*, **401**, 788–791. 10.1038/44565 10548103

[bb22] Lee, J.-W., Park, W. B., Kim, M., Pal Singh, S., Pyo, M. & Sohn, K.-S. (2021). *Inorg. Chem. Front.* **8**, 2492–2504.

[bb23] Lee, J.-W., Park, W. B., Lee, J. H., Singh, S. P. & Sohn, K.-S. (2020). *Nat. Commun.* **11**, 86. 10.1038/s41467-019-13749-3 PMC6941984 31900391

[bb24] Martinetto, P., Blanc, N., Bordet, P., Champdavoine, S., Fabre, F., Guiblain, T., Hodeau, J.-L., Lelong, F., Leynaud, O., Prat, A., Pouyet, E., Uher, E. & Walter, P. (2021). *J. Cult. Herit.* **47**, 89–99.

[bb25] Oliynyk, A. O., Adutwum, L. A., Harynuk, J. J. & Mar, A. (2016). *Chem. Mater.* **28**, 6672–6681.

[bb26] Omori, N. E., Bobitan, A. D., Vamvakeros, A., Beale, A. M. & Jacques, S. D. M. (2023). *Philos. Trans. R. Soc. A*, **381**, 20220350. 10.1098/rsta.2022.0350 PMC10493554 37691470

[bb27] Park, W. B., Chung, J., Jung, J., Sohn, K., Singh, S. P., Pyo, M., Shin, N. & Sohn, K.-S. (2017). *IUCrJ*, **4**, 486–494. 10.1107/S205225251700714X PMC5571811 28875035

[bb28] Poline, V., Bordet, P., Leynaud, O., Prat, A., Bruyère, R., Blanc, N., Lelong, F. & Martinetto, P. (2023). *Eur. Phys. J. Plus*, **138**, 239.

[bb29] Pouyet, E., Miteva, T., Rohani, N. & de Viguerie, L. (2021). *Sensors*, **21**, 6150. 10.3390/s21186150 PMC8471921 34577356

[bb30] Purushottam Raj Purohit, R. R. P., Tardif, S., Castelnau, O., Eymery, J., Guinebretière, R., Robach, O., Ors, T. & Micha, J.-S. (2022). *J. Appl. Cryst.* **55**, 737–750. 10.1107/S1600576722004198 PMC9348891 35974740

[bb31] Rowles, M. R. & Buckley, C. E. (2017). *J. Appl. Cryst.* **50**, 240–251.

[bb32] Scarlett, N. V. Y., Rowles, M. R., Wallwork, K. S. & Madsen, I. C. (2011). *J. Appl. Cryst.* **44**, 60–64.

[bb33] Stock, S. R., Laugesen, M., Birkedal, H., Jakus, A., Shah, R., Park, J.-S. & Almer, J. D. (2019). *J. Appl. Cryst.* **52**, 40–46.

[bb34] Thompson, P., Cox, D. E. & Hastings, J. B. (1987). *J. Appl. Cryst.* **20**, 79–83.

[bb35] Tin Kam Ho, (1998). *IEEE Trans. Pattern Anal. Mach. Intell.* **20**, 832–844.

[bb36] Vamvakeros, A., Coelho, A. A., Matras, D., Dong, H., Odarchenko, Y., Price, S. W. T., Butler, K. T., Gutowski, O., Dippel, A.-C., Zimmermann, M., Martens, I., Drnec, J., Beale, A. M. & Jacques, S. D. M. (2020). *J. Appl. Cryst.* **53**, 1531–1541.

[bb37] Van der Walt, S., Schönberger, J. L., Nunez-Iglesias, J., Boulogne, F., Warner, J. D., Yager, N., Gouillart, E. & Yu, T. (2014). *PeerJ*, **2**, e453. 10.7717/peerj.453 PMC4081273 25024921

[bb38] Wang, B., Guan, Z., Yao, S., Qin, H., Nguyen, M. H., Yager, K. & Yu, D. (2016). *Proceedings of the 2016 New York Scientific Data Summit (NYSDS)*, 14–17 August 2016, New York, NY, USA, pp. 1–5. IEEE.

[bb39] Wang, B., Yager, K., Yu, D. & Hoai, M. (2017). *Proceedings of the 2017 IEEE Winter Conference on Applications of Computer Vision (WACV)*, 24–31 March 2017, Santa Rosa, CA, USA, pp. 697–704. IEEE.

[bb40] Wang, H., Xie, Y., Li, D., Deng, H., Zhao, Y., Xin, M. & Lin, J. (2020). *J. Chem. Inf. Model.* **60**, 2004–2011. 10.1021/acs.jcim.0c00020 32208721

[bb41] Yang, X., De Carlo, F., Phatak, C. & Gürsoy, D. (2017). *J. Synchrotron Rad.* **24**, 469–475. 10.1107/S1600577516020117 28244442

