# Supplementary material for: Neural networks for rapid phase quantification of cultural heritage X-ray powder diffraction data
Source: J Appl Crystallogr. 2024 May 31;57(Pt 3):831–41. doi: 10.1107/S1600576724003704 (PMC11151672; doi:10.1107/S1600576724003704)
Supplement: Supplementary file 1 [file j-57-00831-sup1.pdf]

JOURNAL OF APPLIED CRYSTALLOGRAPHY

Volume 57 (2024)

Supporting information for article:

Neural networks for rapid phase quantification of cultural heritage X-ray powder diffraction data

Victor Poline, Ravi Raj Purohit Purushottam Raj Purohit, Pierre Bordet, Nils Blanc and Pauline Martinetto

Supplementary Information: Neural networks for rapid phase quantification of Cultural Heritage X-ray powder diffraction data

Victor Poline[1], Ravi Raj Purohit Purushottam Raj Purohit[2], Pierre Bordet[1], Nils Blanc[1], and Pauline Martinetto[1]

[1]Univ. Grenoble Alpes, CNRS, Grenoble INP, Institut Néel, 38000 Grenoble
[2]Univ. Grenoble Alpes, CEA, IRIG, MEM, NRS, 17 rue des Martyrs, Grenoble 38000, France

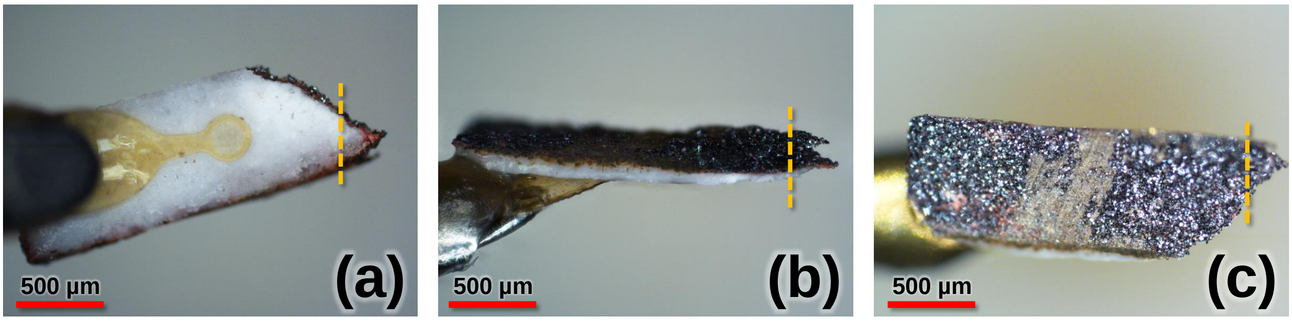

Figure S1 – Photographies of the mock up from the bottom (a), side (b) and top (c). From bottom to top, the layers are made of anhydrite, cinnabar and romarchite. The dashed orange line corresponds to the tomographic slice acquired in XRD-CT during the experiments.

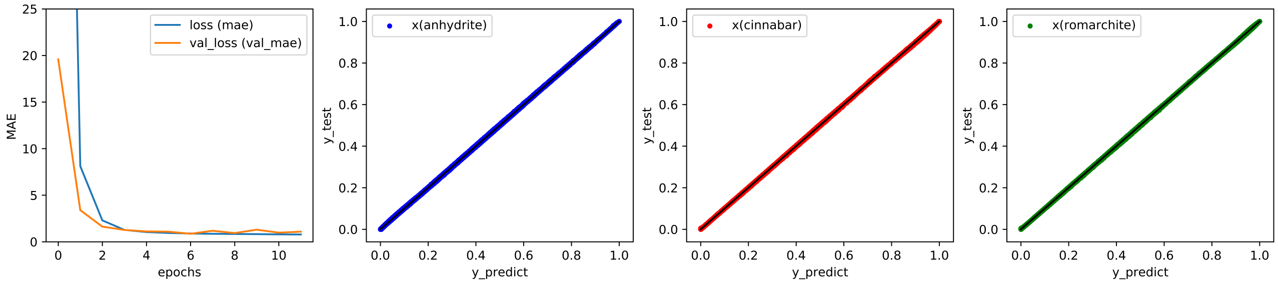

Figure S2 – Summary of the training for phase fraction predictions for a one layer DNN. On the left, the evolution of the mean absolute error as a function of epoch (the y-axis has been partially cut off for the first epoch to improve readability). It should be noted that learning stopped before the end of the 20 epochs because the MAE was not decreasing sufficiently for the DNN to provide better results. The other three graphs represent, for each phase (anhydrite, cinnabar, romarchite), the DNN predictions at the latest epoch compared to the true value of the phase fraction.

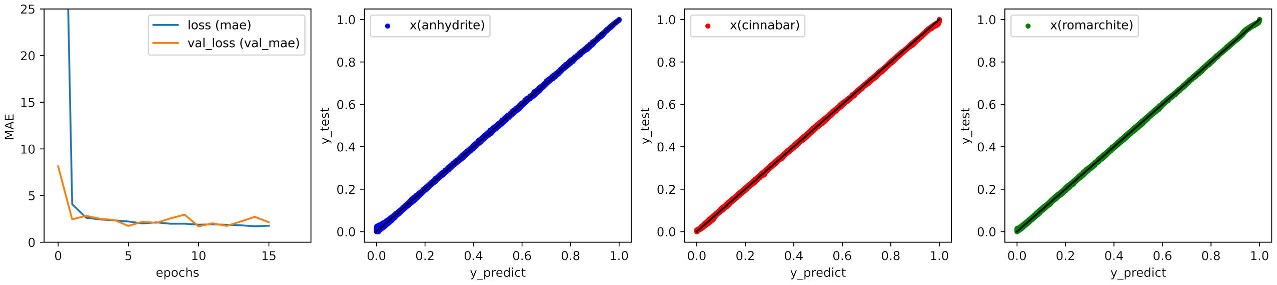

Figure S3 – Summary of the training for phase fraction predictions for a 2 layers DNN. On the left, the evolution of the mean absolute error as a function of epoch (the y-axis has been partially cut off for the first epoch to improve readability). It should be noted that learning stopped before the end of the 20 epochs because the MAE was not decreasing sufficiently for the DNN to provide better results. The other three graphs represent, for each phase (anhydrite, cinnabar, romarchite), the DNN predictions at the latest epoch compared to the true value of the phase fraction.

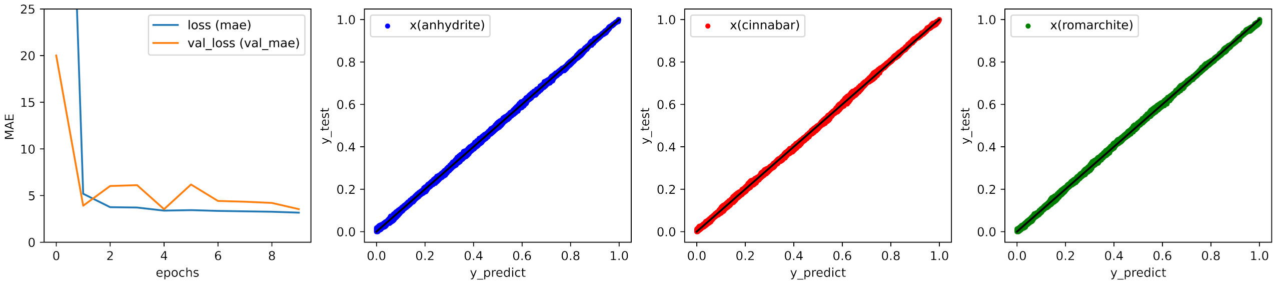

Figure S4 – Summary of the training for phase fraction predictions for a 3 layers DNN. On the left, the evolution of the mean absolute error as a function of epoch (the y-axis has been partially cut off for the first epoch to improve readability). It should be noted that learning stopped before the end of the 20 epochs because the MAE was not decreasing sufficiently for the DNN to provide better results. The other three graphs represent, for each phase (anhydrite, cinnabar, romarchite), the DNN predictions at the latest epoch compared to the true value of the phase fraction.

Table S4 – Summary of the training metrics for different DNN architectures

	1 layer	2 layers	3 layers
MAE	0.8440	1.7677	3.1676
MSE	1.4939	6.1872	19.2908
Training time	2'36"	6'21"	8'08"
Number of epochs	11	16	9

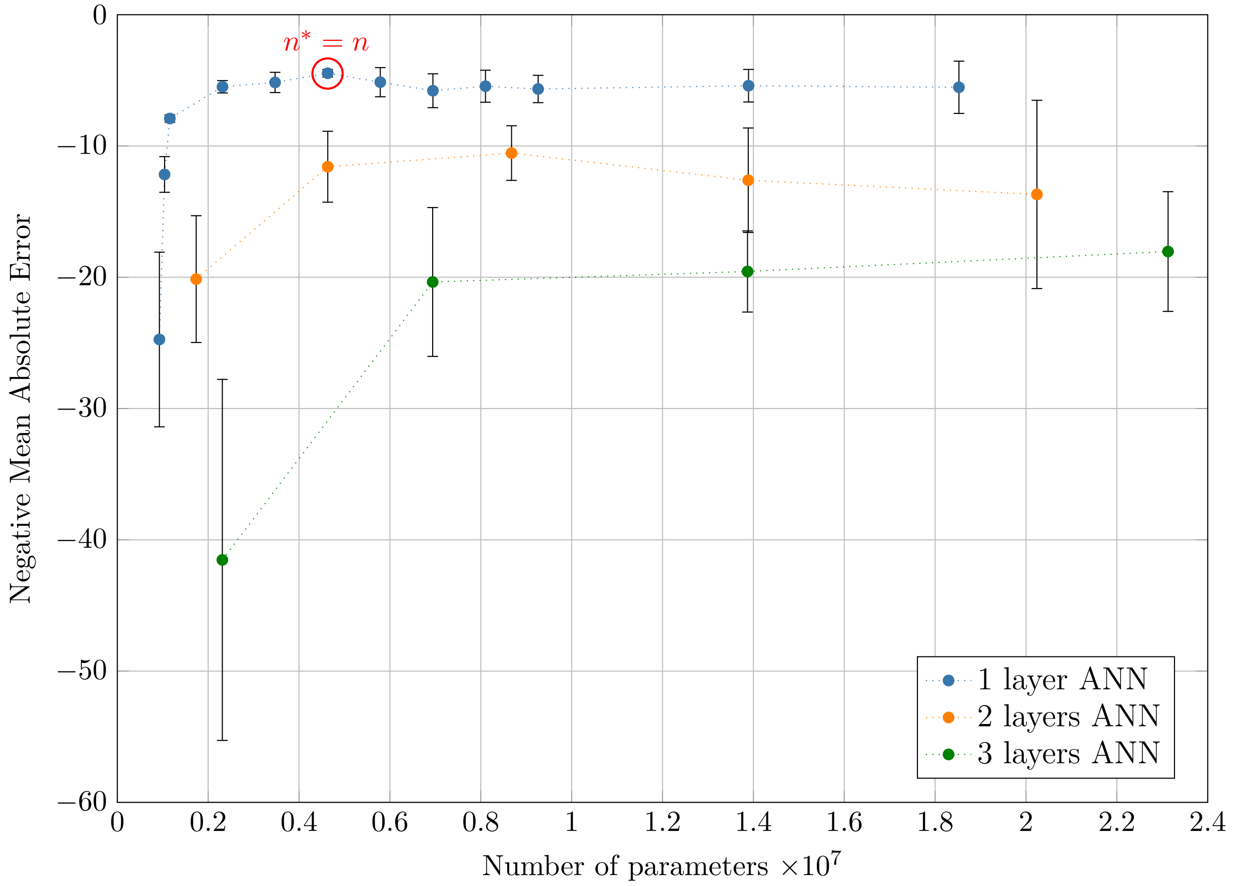

Figure S5 – Evolution of the metric NMAE vs. the number of parameters (weights and biases) of the ANNs for a 5-fold cross-validation on a 400k dataset, error bars are the standard deviation on the 5 folds. Red circle represents the optimum obtained for a 1 layer ANN with a number of neurons n^* = the number of observations in the patterns n. For a 1 layer ANN of an input layer of dimension $n = 2150$, an hidden layer of dimension $n^* = 2150$, and an output layer of 3 neurons, the number of parameters is : $2150 \times 2150 + 2150 + 2150 \times 3 + 3 = 0.4631103 \times 10^7$ parameters.

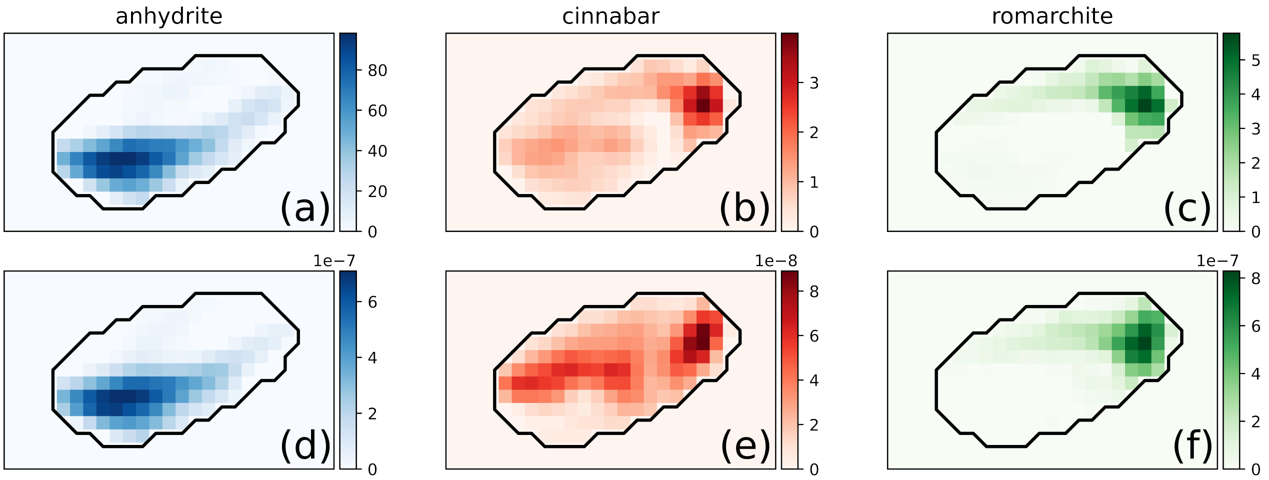

Figure S6 – Phase fraction maps of the 3 phases of the mock-up predicted by the DNN (a, b, c) and by serial Rietveld refinement (d,e,f). The scale is presented without any normalization. The approximate shape of the sample is shown as the black line.

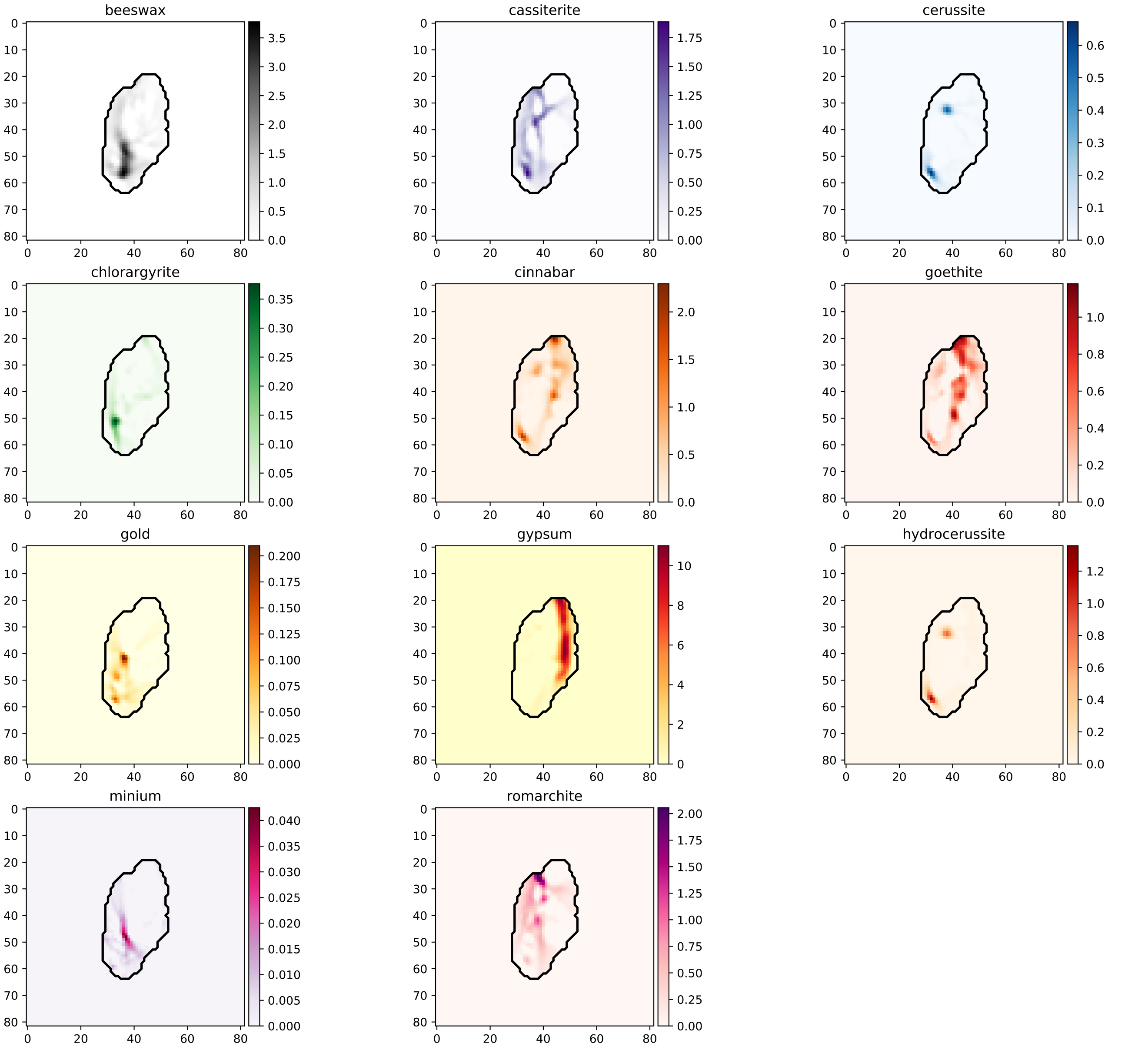

Figure S7 – Phase fraction maps reconstructed from predictions on the sinogram for each phase present in the historical sample. From top left to bottom right : beeswax, cassiterite, cerussite, chlorargyrite, cinnabar, goethite, gold, gypsum, hydrocerussite, minium, romarchite

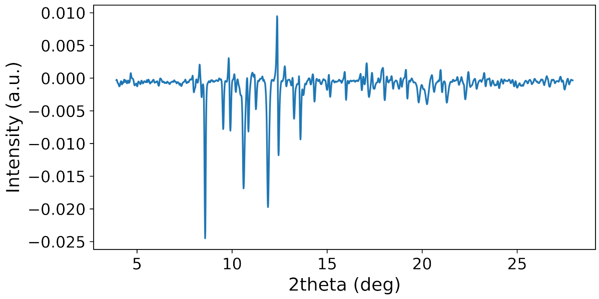

Figure S8 – Example of an anomaly on a reconstructed pattern

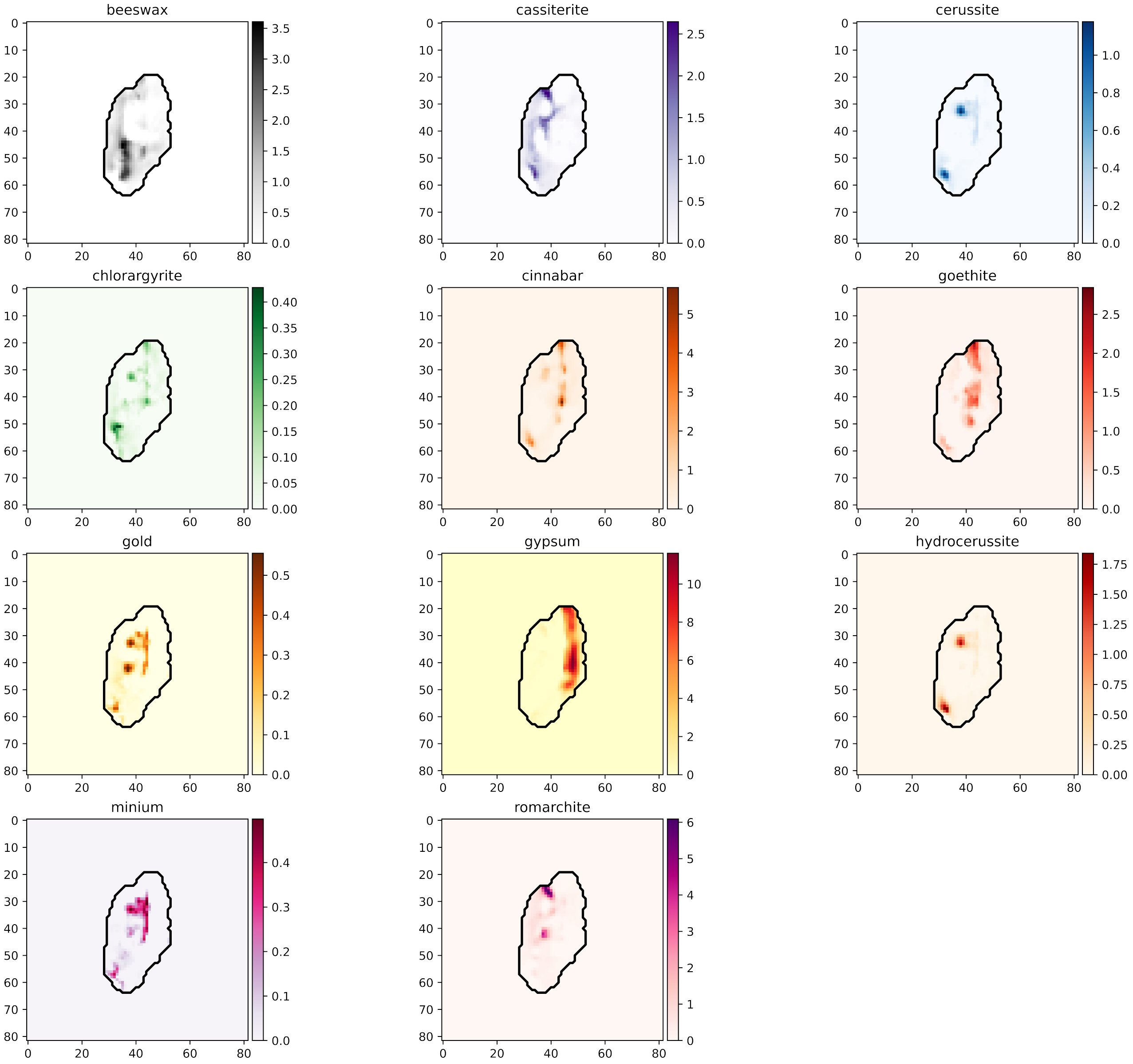

Figure S9 – Phase fraction maps obtained from predictions made on reconstructed patterns. From top left to bottom right : beeswax, cassiterite, cerussite, chlorargyrite, cinnabar, goethite, gold, gypsum, hydrocerussite, minium, romarchite

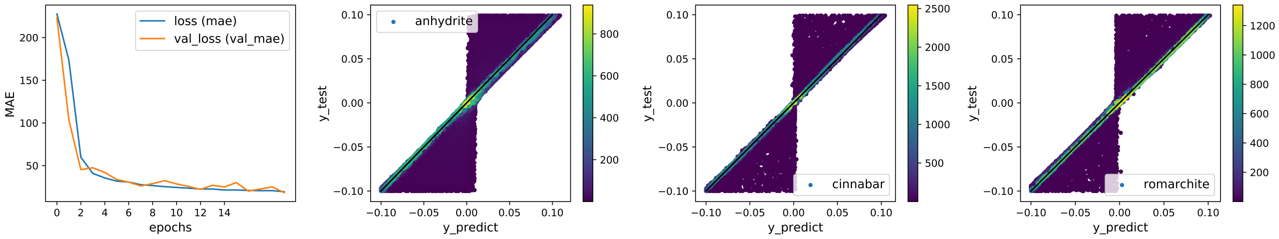

Figure S10 – Summary of the training for phase fraction predictions. On the left, the evolution of the mean absolute error as a function of epoch. It should be noted that learning stopped before the end of the 20 epochs because the MAE was not decreasing sufficiently for the DNN to provide better results. The other three graphs represent, for each phase, the DNN predictions at the latest epoch compared to the true value of the phase misplacement (density plot for better readability).

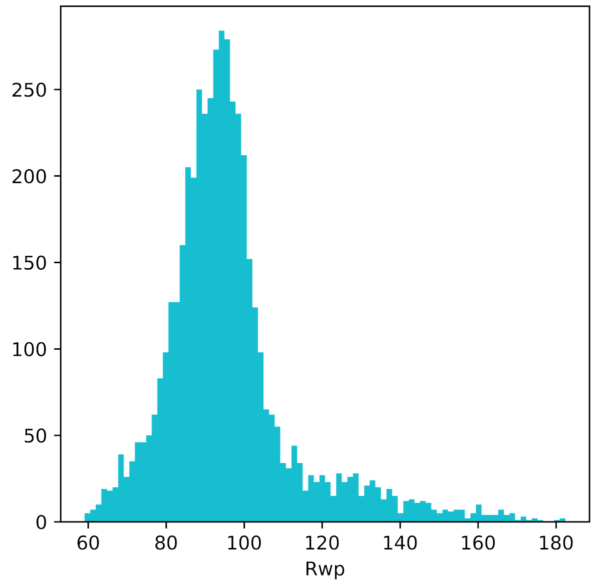

Figure S11 – Histogram representation of *Rwp* for all the patterns in the mock-up.

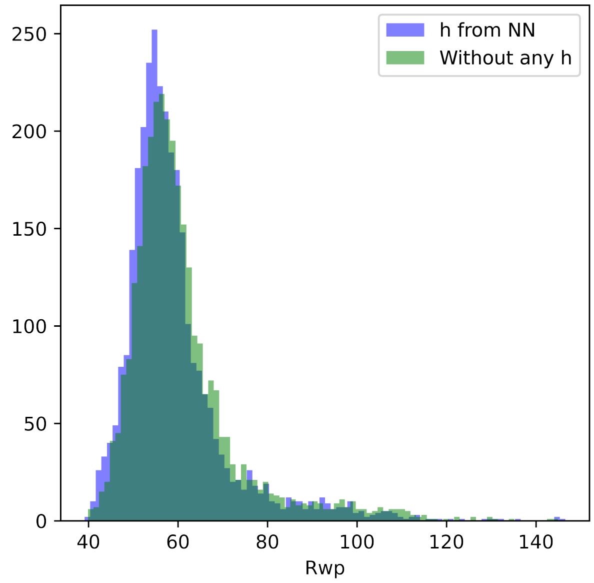

Figure S12 – Comparison between *Rwp* histograms with sample displacements from NN predictions vs no sample displacements for the historical sample.

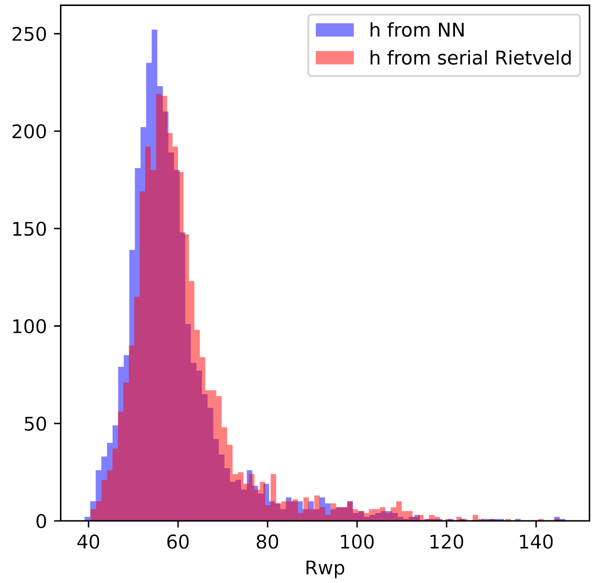

Figure S13 – Comparison between *Rwp* histograms with sample displacements from NN predictions vs sample displacements from serial Rietveld refinement for the historical sample.
